# The effects of type 2 diabetes mellitus on the corneal endothelium and central corneal thickness

**DOI:** 10.1038/s41598-021-87896-3

**Published:** 2021-04-15

**Authors:** Yoo Jin Kim, Tae Gi Kim

**Affiliations:** Department of Ophthalmology, Kyung Hee University Hospital at Gangdong, Kyung Hee University, # 892, Dongnam-ro, Gangdong-gu, Seoul, 05278 Republic of Korea

**Keywords:** Epidemiology, Diseases, Endocrinology, Medical research, Risk factors

## Abstract

Aim of this study is to evaluate the differences in corneal endothelial cell morphology and corneal thickness in patients with and without type 2 diabetes related to age, disease duration, and HbA1c percentage. This retrospective cross-sectional study included 511 (1022 eyes) type 2 diabetes patients and 900 (1799 eyes) non-diabetic patients. The endothelial cell density (ECD), variation in endothelial cell size (CV), percentage of hexagonal cells, and central corneal thickness (CCT) were analyzed using a noncontact specular microscope and a Pentacam Scheimpflug camera. We also examined the correlation between the corneal parameters and the duration of diabetes. For total ages, the subjects with type 2 diabetes showed significantly lower ECD, hexagonality, higher CV, and thicker CCT than the control group. This difference was more pronounced in patients with long-standing DM (≥ 10 years) and high HbA1c (≥ 7%). When stratified by age group, from the 60 s group, corneal endothelial cell parameters showed a statistically significant difference between DM and control groups. The duration of diabetes was inversely correlated with ECD (*r* =  − 0.167; *p* = 0.000). These findings suggest that diabetes affects corneal endothelial cell in older age and those with long-standing DM and higher HbA1c. Regular corneal endothelial examinations are required in diabetic patients.

## Introduction

The corneal endothelium is a single layer of cells that plays a major role in maintaining the optical transparency of the cornea through the Naþ/Kþ-ATPase pump activity^1^. Corneal endothelial cells have the highest density at birth, which decreases by approximately 0.5% per year following aging^2^. Factors other than aging that affect corneal endothelial cell loss include genetics, race, trauma, intraocular surgery, and infection^3–8^.

Diabetes mellitus (DM) is a metabolic disease characterized by chronic hyperglycemia resulting from dysfunctional insulin secretion or insulin action. Chronic hyperglycemia may lead to micro- and macrovascular disorders, and it causes changes in almost all ocular structures. Diabetic retinopathy is the most common ophthalmic complication of diabetes, but diabetes can also affect the cornea^9^.

There is extensive literature on the association between diabetes and corneal endothelial cells^10–15^. The conclusions of previous studies on the effect of DM on the corneal endothelium are inconsistent. In previous studies that compared type 1 and type 2 DM patients with normal subjects, corneal endothelial cell density (ECD) was reported to decrease in both DM group^12^. However, some studies reported that there was no difference between type 2 DM patients and normal subjects^13^. Lee et al.^14^ reported that patients with diabetes that had lasted for 10 years had more corneal morphological abnormalities regardless of the type of diabetes. On the other hand, Choo et al.^11^ reported that ECD decreased in diabetes, but they did not show any difference in coefficient of variation (CV), hexagonality, and central corneal thickness (CCT) in type 2 DM. Storr-Paulsen et al.^15^ reported that CCT was significantly increased in the diabetic group, and higher HbA1c was associated with lower ECD in type 2 DM.

As indicated above, the changes in corneal endothelial cells in type 2 DM are still controversial different from those of type 1 DM. Most studies had a sample size of less than 100 cases, leading to high variability in results. In addition, a few studies comparatively analyzed age groups for the impact on corneal endothelial cells. Sudhir et al.^10^ compared 1191 patients with diabetes stratified by age but only enrolled 120 healthy subjects in their study. Su et al.^16^ enrolled 3239 patients with diabetes but only analyzed CCT, not corneal endothelial cells, and did not perform comparative analysis by age group.

The aim of the present study was to compare corneal endothelial cell characteristics, such as ECD, CV, hexagonality, and CCT, of patients with type 2 diabetes and healthy controls stratified by age group in a large sample. In addition, the correlations between the duration of diabetes, HbA1c, and corneal endothelial cell morphology were also analyzed.

## Results

A total of 1411 patients were included in the study: 1022 eyes in 511 patients (264 males, 247 females, mean age 65.6 ± 11.1 years) were included in the DM group, and 1799 eyes in 900 patients (333 males, 567 females, mean age 67.9 ± 11.2 years) were included in the control group. All patients were Asians. The mean duration of the DM was 10.8 ± 8.7 years, and the mean level of HbA1c level was 7.54 ± 1.78% in the DM group. There were no differences between the groups in terms of age. The demographics and age distribution of the DM and control groups are shown in Table 1.

**Table 1:** Demographics and age distributions of diabetic and control groups.

	DM group	Control group	*p* value*
Sample size (eyes)	511 (1022)	900 (1799)	–
Sex (M:F)	264:247 (528:494)	333:567 (666:1133)	
Age, year (mean ± SD)	65.6 ± 11.1	67.9 ± 11.2	0.637
HbA1c (mean ± SD)	7.54 ± 1.78	–	–
Duration of diabetes (years) (mean ± SD)	10.8 ± 8.7	–	–
Age distribution (eyes)			–
40–49	92	115	–
50–59	163	182	–
60–69	378	650	–
70–79	302	606	–
> 80	87	246	–

*p value by independent t-test.

### Comparative analysis of corneal parameters in all age group

For all ages, the corneal parameters in the DM and control groups were significantly different. (Table 2). The ECD and hexagonality were significantly lower in the DM group than in the control group (*p* = 0.023 and 0.030, respectively), and CV and CCT were significantly greater in the DM group (*p* = 0.000 and 0.000, respectively).

**Table 2:** Comparison of the mean values of ECD, CV, hexagonality, and CCT of diabetic eyes and controls across all ages (mean ± SD).

	DM group	Control group	p*
ECD, cells/mm^2^	2656.42 ± 379.21	2688.77 ± 351.96	0.023
Coefficient of Variation (%)	36.09 ± 8.00	34.92 ± 7.16	0.000
Hexagonal cell ratio (%)	53.55 ± 9.80	54.41 ± 10.39	0.030
CCT (μm)	551.80 ± 34.10	542.63 ± 33.79	0.000

*ECD* Endothelial cell density, *CV* Coefficient of variation, *CCT* Central corneal thickness.

*p value by independent t-test.

**Figure 1:**
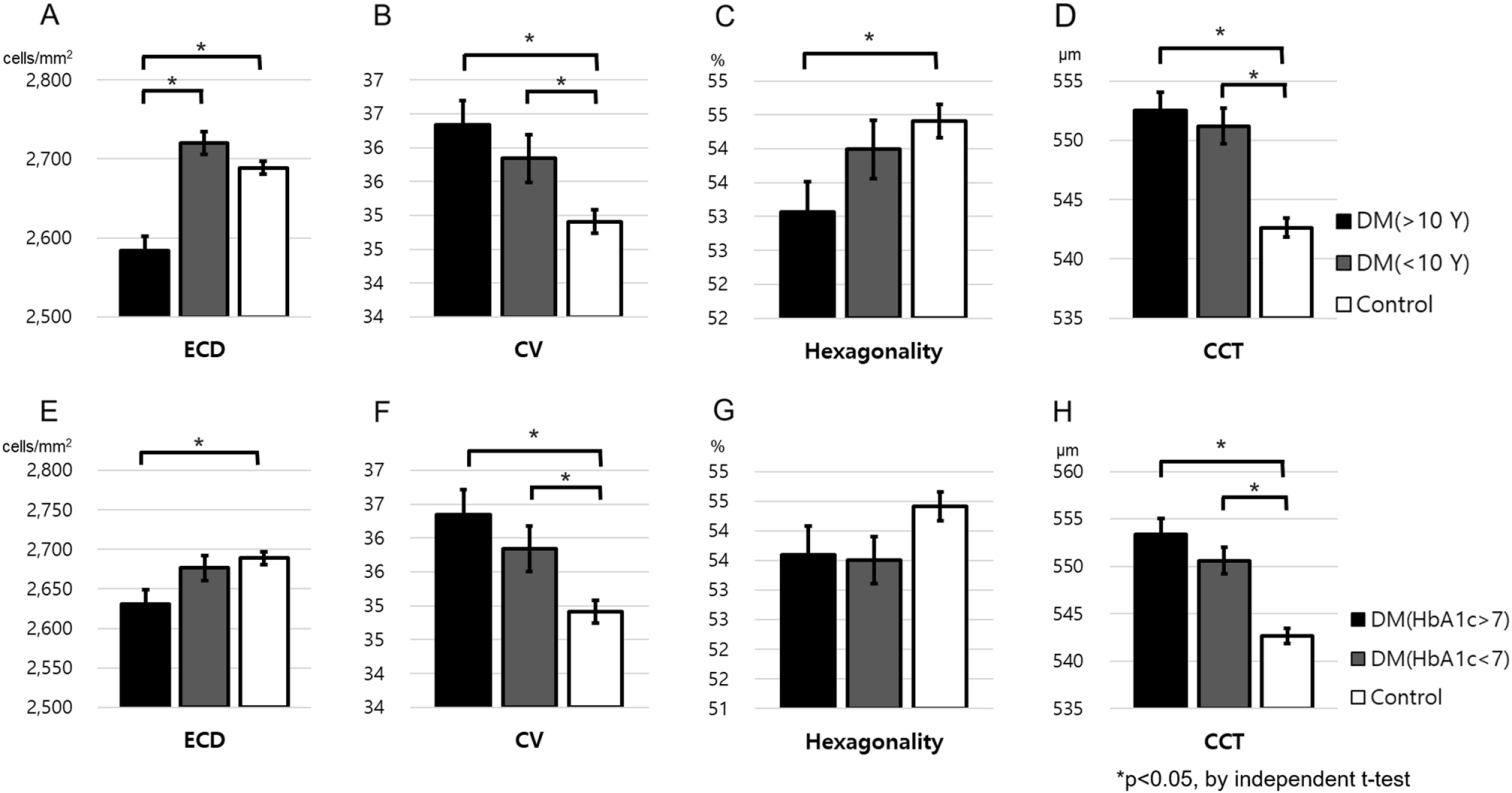
Comparison of endothelial cell parameters of diabetic patients and controls based on diabetic duration and HbA1c% across all ages. (**A**–**D**) Corneal endothelial cell parameters based on disease duration. (**E**–**H**) Corneal endothelial cell parameters based on HbA1c concentration. *ECD* Endothelial cell density, *CV* Coefficient of variation, *CCT* Central corneal thickness.

When analyzed according to the duration of diabetes, the ECD, CV, hexagonality, and CCT of the group of patients with DM lasting 10 years or more were significantly different from those of the control group (Fig. 1A–D). However, in the group of patients with DM lasting for less than 10 years, only CV and CCT were significantly different from those of the control group. Only ECD was significantly different in the long duration (≥ 10 years) and short duration (< 10 years) groups.

When analyzed according to HbA1c, in high HbA1c group (≥ 7%), ECD, CV, and CCT were statistically significantly different compared to the control groups. Meanwhile, in the group with HbA1c < 7%, only CV and CCT were significantly different (Fig. 1E–H). There was no statistically significant difference between the groups with HbA1c of ≥ 7 and < 7%.

### Comparative analysis of corneal parameters by age group

**Figure 2:**
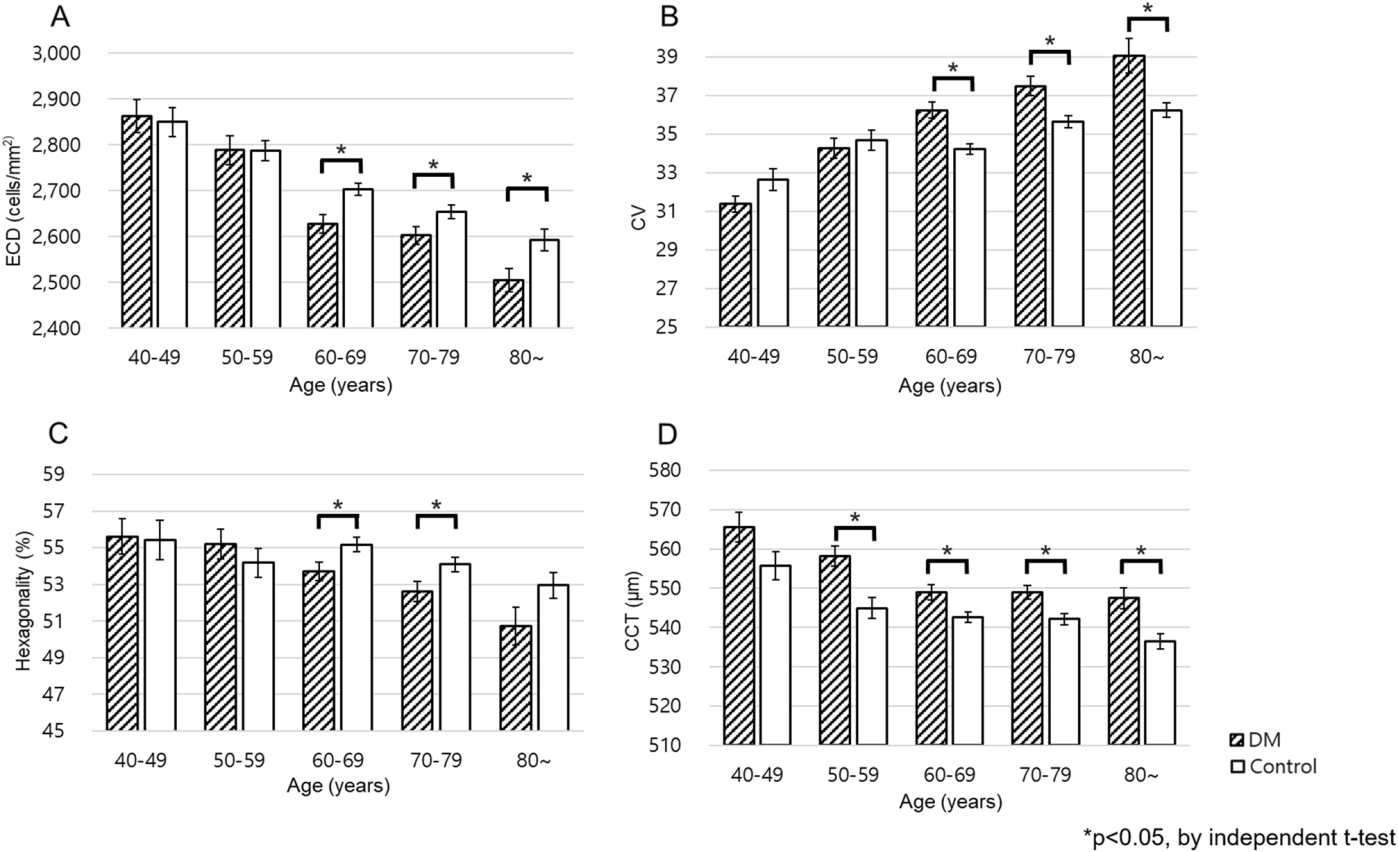
Comparison of endothelial cell parameters of diabetic patients and control based on age. (**A**) Mean ECD, (**B**) mean CV, (**C**) mean hexagonality and (**D**) mean CCT across the age groups. *ECD* Endothelial cell density, *CV* Coefficient of variation, *CCT* Central corneal thickness.

ECD, CV, and hexagonality of the DM and control groups stratified by age were significantly different after 60 years, and CCT showed a statistically significant difference after 50 years. (Fig. 2).

**Figure 3:**
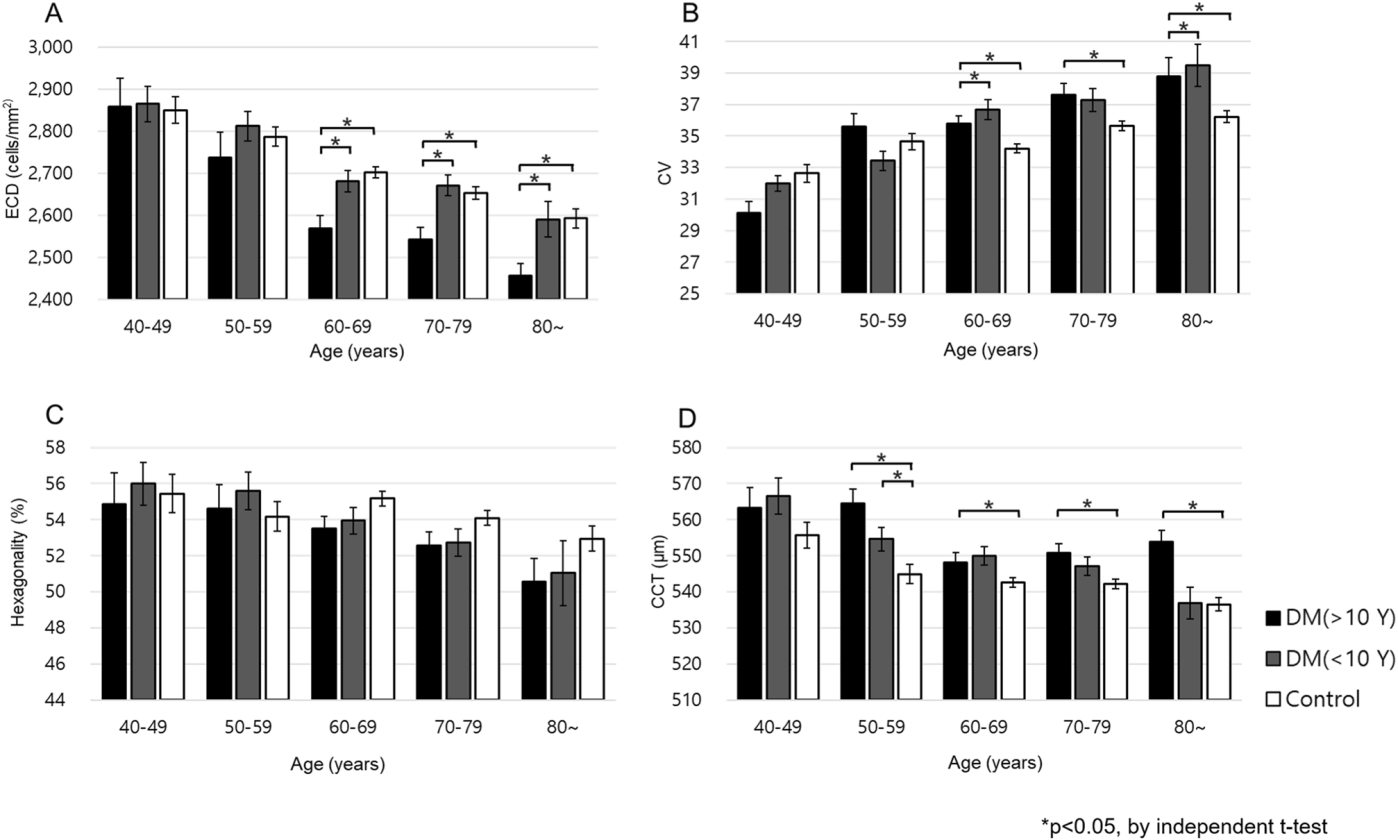
Comparisons of the mean values of ECD (**A**), CV (**B**), hexagonality (**C**) and CCT (**D**) of the diabetic groups based on diabetic duration across the age groups. *ECD* Endothelial cell density, *CV* Coefficient of variation, *CCT* Central corneal thickness.

The comparison of corneal parameters based on the duration of disease showed differences related to age group. ECD and CV after 60 years and CCT after 50 years in the group with a long duration of disease (≥ 10 years) were significantly different from the control group. Hexagonality showed a significant difference only for ages between 60 and 69 years (Fig. 3). Between the long duration group (≥ 10 years) and the short duration group (< 10 years), only ECD showed a statistically significant difference after age 60 (Fig. 3A).

**Figure 4:**
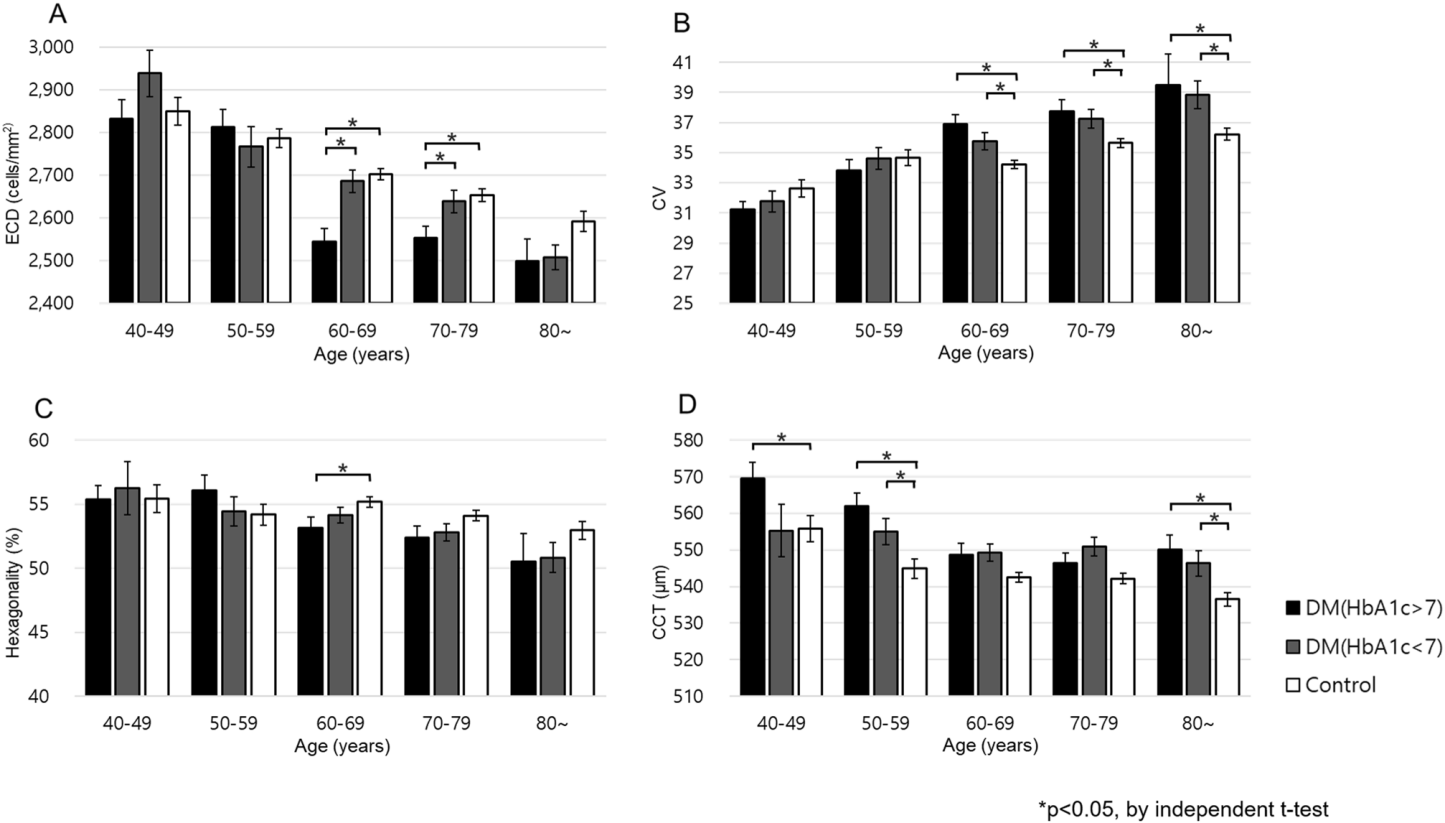
Comparisons of the mean values of ECD (**A**), CV (**B**), hexagonality (**C**) and CCT (**D**) of the diabetic groups based on HbA1c% across all age groups. *ECD* Endothelial cell density, *CV* Coefficient of variation, *CCT* Central corneal thickness.

In the high HbA1c group (≥ 7%), the ECD at ages between 60 and 79 years were significantly lower than those of the control group (*p* = 0.000 and 0.002, respectively), and CV was statistically different from that of the control group after 60 years of age. (*p* < 0.05) (Fig. 4A,B). There were differences in CCT at ages between 40 and 59 years (*p* = 0.017 and 0.000, respectively) and hexagonality at ages between 60 and 69 years (*p* = 0.025), however, there was no clear tendency related to aging (Fig. 4C,D). In the low HbA1c group (< 7%), only CCT in the 50 s and 80 s and CV at ages between 60 and 79 years were significantly different from those of the control group (Fig. 4B,D). Only ECD in the high HbA1c (≥ 7%) and low HbA1c (< 7%) groups showed a statistically significant difference at ages between 60 and 69 years (*p* = 0.001) (Fig. 4A).

### Pearson correlation analysis

Pearson correlation analysis showed that age had significant correlations with ECD, CV, hexagonality, and CCT. In addition, DM duration had significant correlations with ECD. (Table 3.) However, the HbA1c levels had no significant correlations with ECD, CV, hexagonality, and CCT.

**Table 3:** Correlations between endothelial cell parameters and age, diabetic duration, and HbA1c% in the diabetic group.

	ECD	CV	Hexagonality	CCT
**Age**				
R value	−0.257	0.252	−0.136	−0.141
*p* value	0.000	0.000	0.000	0.000
**DM duration**				
R value	−0.167	0.059	−0.059	0.020
*p* value	0.000	0.057	0.060	0.535
**HbA1c**				
R value	0.064	−0.051	−0.010	0.057
*p* value	0.058	0.130	0.765	0.096

*ECD* Endothelial cell density, *CV* Coefficient of variation, *CCT* Central corneal thickness.

*p* value by Pearson’s correlation coefficient test.

## Discussion

DM affects all structural layers of the cornea, including the corneal epithelium, corneal nerves, tear film, and corneal endothelium^17^. Clinically observed corneal diabetic complications include recurrent epithelial erosion, delayed wound repair, neurotrophic ulcers, and decreased corneal sensitivity. Morphological and functional changes also occur in the corneal endothelium, and diabetes carries an increased risk of endothelial complications after intraocular surgery. Although the pathophysiological mechanisms underlying diabetic damage of the corneal endothelium are not yet clear, osmotic damage due to excessive sorbitol accumulation, oxidative damage due to glycation end-product accumulation, and the reduction of corneal endothelial cell adhesion due to direct adhesive protein modifications of Descemet's membrane by AGEs has been proposed^18–20^.

It is clinically important to analyze the corneal endothelial cell status in patients with type 2 diabetes. Corneal endothelial cells can decrease with surgical trauma, such as through cataract surgery. In patients with diabetes, preoperative corneal endothelial cell dysfunction may cause more corneal endothelial cell damage postoperatively. In addition, corneal endothelial cell loss postoperatively may cause corneal decompensation, highlighting the importance of considering corneal endothelial cell transplantation. In addition, for corneal donor patients with diabetes, the possibility of deterioration of corneal endothelial cell function should be considered. Particularly, during Descemet membrane endothelial keratoplasty (DMEK) preparation, the cornea of a donor with DM has a high probability of failure during DMEK preparation because corneal endothelial cells are firmly attached to the posterior stroma^21–23^. Therefore, when the eye bank is planning DMEK, for donors with DM, morphological changes in the corneal endothelium and difficulty in DMEK preparation should be considered.

Several studies have evaluated the endothelium in patients with type 2 diabetes and normal subjects. Most of them agree that the corneal endothelium of diabetic patients changes morphologically^10–16,24–26^. Su et al.^16^ reported that diabetics had thicker corneas and Roszkowska et al.^26^ reported a significant decrease in cell density in type 2 diabetic patients. Other studies also showed a significant decrease in ECD and hexagonality and an increase in CV in type 2 DM patients^12,14^. However, several studies found no difference between the ECDs of diabetics and normal subjects^27,28^. The effect of DM on the corneal endothelium remains controversial. The high variability of study results may attributable to a small sample size of approximately 100 cases. In contrast, our study had a large sample size of more than 500 participants in both type 2 diabetes and control groups in addition to subgroup analyses according to the age group and severity and duration of DM.

In the present study, it was detected that ECD, CV, hexagonality, and CCT showed significant differences between long durations of diabetes (≥ 10 years) and control in all age (Fig. 1) However, only CV and CCT showed significant differences between short durations of diabetes (< 10 years) and control. In addition, high HbA1c (≥ 7%) patients showed differences in ECD, CV, and CCT, and low HbA1c patients (< 7%) showed only differences in CV and CCT. Therefore, it can be assumed that the long-standing diabetic patients had a greater effect on corneal endothelial change than in high Hb1Ac patients. Previous studies also reported that changes in ECD, CV, and CCT are associated with the duration of DM, and patients who had had DM for more than 10 years had more significant changes^14,29^. Corneal endothelial cell change in diabetes is a chronic microvascular complication, and complications occur more frequently when DM is prolonged and patients are chronically exposed to hyperglycemic conditions^30^. On the other hand, HbA1c reflects short-term DM control, and it is reported that CCT may change within a short period depending on the HbA1c level^14^. This suggests that the duration of diabetes is more strongly associated with chronic corneal endothelial damage than HbA1c. In the correlation analysis, DM duration correlated with ECD; however, HbA1c showed no significant correlation with corneal parameters. This is because HbA1c reflects short-term diabetic control instead of chronic corneal endothelial cell damage. Therefore, the duration of diabetes is recommended over HbA1c for predicting corneal endothelial cell damage.

Moreover, chronic exposure to hyperglycemic conditions can cause microvascular complications as well as diabetic neuropathy due to nerve damage. Diabetic nerve damage results in trigeminal nerve abnormalities and diabetic corneal neuropathies, which can affect the cornea. According to previous studies, central corneal sensitivity and corneal nerve length may decrease in the early stages of diabetes^31^. In addition, it has been reported that trigeminal neuropathy causes disorders in reflex lacrimation, causing tear film instability, resulting in dry eye syndrome^32^. Furthermore, it mainly causes corneal epithelial cell damage; however, the direct correlation between corneal endothelial cell changes and diabetic corneal neuropathy is unclear. Recently, dell'Omo et al. reported that there was no correlation between corneal endothelial cells and corneal neuropathy^33^. The reason for such results is explained by the fact that neuropathy is an early change in diabetes, whereas corneal endothelial cell dysfunction is a chronic change^34,35^.

When the groups were stratified by age, ECD, hexagonality, and CCT decreased whereas CV tended to increase with aging, which was consistent with the findings of previous studies^10,36^. A few papers have compared diabetic patients by dividing them into age groups. In 2012, Sudhir et al.^10^ analyzed the data of 1191 DM patients and 120 non-diabetic patients stratified by age. They reported that CV and hexagonality were greater in diabetic patients than in controls aged between 50 and 69 years, and CCT was thicker in diabetic patients than in controls aged between 60 and 69 years. Contrary to a study by Sudhir et al.^10^, our study had more than 500 patients in the control group. To our knowledge, our study has had the largest sample size for the control group among all studies comparing corneal endothelial cell morphology between type 2 diabetes and control groups. In this study, most corneal parameters significantly differed in DM and control groups after the age of 60 years. The reason for the pronounced difference between the DM and control groups as the age increased is presumed to be because the DM duration is more likely to become longer as the elderly age, and the older person is vulnerable to corneal endothelial cell damage.

When the DM duration and HbA1c of the DM and control groups stratified by age were analyzed, CCT showed a significant difference after 50 years in patients with long-standing diabetes (≥ 10 years) and ECD, CV, and hexagonality showed significant differences after 60 years. Similar trends were observed in the high HbA1c group (≥ 7%); CCT showed a difference at the age of 40 years, whereas ECD, CV, and hexagonality showed significant differences at the age of 60. The reason that differences in CCT at an early age of 40 years in patients with elevated HbA1c may be the short-term impact of HbA1c on CCT.

The limitation of this study was the cross-sectional study design and lack of confirmation of prospective changes. In future studies, consecutive changes in corneal parameters with time should be analyzed. Second, compared to previous studies, although the sample size was relatively large, the generalizability of study results remained limited. Therefore, hexagonality and CCT did not show a clear trend with age in the subgroup analysis. Finally, the HbA1c test was not performed for controls. Despite these limitations, our study has advantages of the relatively large sample of more than 1,000 patients, subdivided into age groups. Furthermore, subgroup analyses according to the age, HbA1c level, and disease duration were performed.

In conclusion, the corneas of patients with type 2 DM showed an increase in thickness and CV and reductions in ECD and hexagonality compared with the healthy controls. These changes were more in older type 2 DM patients (≥ 60 years) with a long duration of disease (≥ 10 years) and high HbA1c (≥ 7%). In the correlation analysis, age and the duration of DM affected ECD. These results are from a large population-based sample, and which support the theory that type 2 DM can affect corneal endothelial cells. Therefore, it is necessary to evaluate not only the retina but also the corneal endothelial cells regularly during follow-up for type 2 DM patients.

## Methods

### The study population

This cross-sectional study complied with the Declaration of Helsinki, and it was approved by the Institutional Review Board of Kyung Hee University Hospital at Gangdong (approval number: 2020-10-016). An exemption was granted from the requirement for informed consent by the Institutional Review Board of Kyung Hee University Hospital at Gangdong because the study had a retrospective design. Patients over 40 years old age, who visited our outpatient clinic between 2016 and 2019 and underwent specular microscopy were included. The diagnosis of type 2 diabetes and the duration of diabetes were analyzed based on medical history, and all the diabetic patients were taking oral or parenteral antidiabetic medication. In the DM group, the serum glycosylated hemoglobin (HbA1c) levels were analyzed within 2 months before and after ophthalmic visits. The patients underwent corneal specular microscopy, slit-lamp examination, fundus examination, and corneal topography. The exclusion criteria included glaucoma, uveitis, intraocular surgery or laser treatment, previous use of contact lenses, and history of corneal diseases such as keratoconus, Fuchs endothelial dystrophy, and corneal opacities.

### Analysis of corneal endothelial cells and central corneal thickness

To assess the corneal endothelial status, the central ECD (cells/mm^2^), variations in the size of endothelial cells (CV) (%), and the percentage of hexagonal cells (%) were analyzed using Topcon SP3000P non-contact endothelial specular microscope (Topcon Corporation, Tokyo, Japan). The subjects were asked to look at the central fixation target, and the auto-alignment function was used. The central endothelial cell density (cells/mm^2^), CV, and the percentage of hexagonal cells were calculated using the software of the specular microscope. CCT was obtained using a non-contact Pentacam rotating Scheimpflug camera (Oculus, Wetzlar, Germany). During the measurements, the subjects fixated on a distant target. The internal software (Pentacam Basic software) automatically determined corneal thickness.

### Statistical analysis

The ECD, CV, hexagonlaity, and CCT of the diabetic patients and control subjects were compared using the independent samples *t*-test in SPSS version 18.0 software (SPSS Inc., Chicago, USA). A Pearson correlation test was performed to determine the relationships between corneal changes, DM duration, and HbA1c%. A p-value of less than 0.05 was considered to be statistically significant.
